# Storage and Ripening Monitoring of Pecorino Cheese Through 2D ^1^H-NMR Relaxation and ANOVA Simultaneous Component Analysis (ASCA): A Comparison with DSC and ATR-FTIR Characterization

**DOI:** 10.3390/molecules30142916

**Published:** 2025-07-10

**Authors:** Francesca Di Donato, Francesco Gabriele, Alessandra Biancolillo, Cinzia Casieri, Angelo Antonio D’Archivio, Nicoletta Spreti

**Affiliations:** Department of Physical and Chemical Sciences, University of L’Aquila, Via Vetoio snc-Coppito, I-67100 L’Aquila, Italy; didonatofrancesc@gmail.com (F.D.D.); francesco.gabriele@univaq.it (F.G.); cinzia.casieri@aquila.infn.it (C.C.); nicoletta.spreti@univaq.it (N.S.)

**Keywords:** cheese, proton magnetic relaxation times correlation, Calorimetry, Infrared Spectroscopy, analysis of variance, simultaneous component analysis

## Abstract

In food processing, non-destructive and non-invasive characterization is a powerful tool for monitoring processes and controlling quality. Cheeses consist of a large variety of products whose nutritional and sensory properties depend on the source materials, cheesemaking procedures, and biochemical transformations occurring during maturation and storage. In this study, proton magnetic resonance relaxation time correlation maps (2D ^1^H-NMR T_1_–T_2_) are used to investigate the effect of the ripening degree on Pecorino cheese and evaluate its evolution during storage in a refrigerator under vacuum-packaging conditions. NMR relaxometry has allowed for non-invasive monitoring of packaged Pecorino cheese slices, and the results were compared with those obtained with the two widely used techniques, i.e., Differential Scanning Calorimetry (DSC) and Attenuated Total Reflectance Fourier-Transform Infrared Spectroscopy (ATR-FTIR). The analysis of variance and simultaneous component analysis (ASCA), separately applied to 2D ^1^H-NMR T_1_–T_2_ correlation maps, DSC, and ATR-FTIR data, suggests that the results obtained with the NMR approach are consistent with those obtained using the two benchmark techniques. In addition, it can distinguish cheeses stored for different durations (storage time) irrespective of their original moisture content (ripening degree), and vice versa, without opening the vacuum-package, which could compromise the integrity of the samples.

## 1. Introduction

In food processing, real-time and non-invasive characterization of the material stream is a powerful tool for process monitoring and quality control. Numerous transformations can occur at different stages of the manufacturing system due to the chemical processes involved in the production and storage of a food commodity. Cheeses, in particular, consist of a large variety of products whose nutritional and sensory properties are not only dependent on the source materials (kind of milk, rennet, and microbic starters) and cheesemaking variables (milk processing, cooking temperature, pH, curd cutting, and salting) but also on the biochemical transformations occurring in the successive maturation process, all of which need to be monitored with designed applications [1,2]. In general, the cheese-making process starts with the formation of a 3D network due to the aggregation of casein molecules, in which fat globules and part of the milk whey are entrapped. Triacylglycerols, contained in the micrometric-sized fat globules of native milk, represent the main fat component of dairy products. Considering microstructure, cheese can therefore be regarded as a complex porous medium where both the confined liquid-like fat and the entrapped aqueous-phase experience restricted dynamics [3]. Nevertheless, the biological transformations (primarily proteolysis, glycolysis, and lipolysis) occurring during maturation [2,4], apart from changing the chemical composition and sensorial attributes of cheese, modify the nature of protein–protein, protein–water, and protein–fat interactions which subsequently has a deep impact on its microstructure and texture, and, consequently, on the dynamics of the confined fat and water molecules [5,6]. Although each type of cheese has its own features in terms of composition and texture depending on the composition of the original milk and manufacturing process, ripening is associated with a loss of moisture and a tightening of the cheese protein structure due to proteolytic processes that convert caseins into a range of large- and intermediate-sized peptides and successively into shorter peptides and amino acids [7,8]. A further consequence of casein hydrolysis is the formation of ionic-charged proteolytic residues that make protein strands and peptides more and more hydratable as ripening progresses [5].

Regarding the fat component, proteolysis of the fat globule membrane causes the disruption of the globules and the formation of irregular pools of non-globular fat confined in voids within the protein matrix [9,10]. Moreover, triacylglycerols no longer protected by the globule membrane are subjected to lipolysis, generating free fatty acids [11] which are precursors of a range of volatile compounds, including methyl ketones, esters, secondary alcohols, lactones, and alkanes, responsible for the cheese flavoring [12].

Nuclear magnetic resonance (NMR) has become an important tool in studying molecules bearing protons in a liquid-phase confined in a wide class of porous materials, ranging from model and biological systems to building materials [13]. The peculiar aspects that make NMR sensitive to the confined state of a liquid are based on the difference in dynamics and magnetic interactions that molecules experience in the proximity of a chemical and/or physical wall with respect to their bulk condition.

By utilizing water protons as NMR-sensitive nuclei, the longitudinal (T_1_) and transverse (T_2_) relaxation of liquid water in confined geometry scales up to three orders of magnitude with respect to bulk water. Then, it is possible to obtain information about the pore-size distribution through the distribution of relaxation times, even if major assumptions are usually required [13]. The local magnetic field gradients found due to the susceptibility differences between fluid and solid matrices of a porous media enhance through the diffusion term the dephasing of the transverse coherence, resulting in T_1_/T_2_ ratios slightly above unity. When paramagnetic impurities are possibly present, a further phase dispersion is produced and the T_1_/T_2_ ratios can become much higher.

Although the T_2_ and/or T_1_ processes have been used for studying the effects of pH on the casein gel structure [14], the addition of emulsifying salt [15], heating [16], and the different processing stages in the manufacture of imitation cheese [17], the NMR relaxometry technique has often not been performed to its fullest possibilities. The NMR signal, where the proton magnetic resonance relaxation times are simultaneously collected and correlated (2D ^1^H-NMR T_1_–T_2_) [18] allows us to effectively separate NMR signals associated with different proton phases. This experimental approach offers typical low-resolution NMR information but enhances accuracy in evaluating phase distribution and exchange regimes. Hürlimann and Song [3,19] have pioneered the application of 2D ^1^H-NMR T_1_–T_2_ to food-related issues, particularly in dairy products. Recently, this technique has been employed to monitor the maturation of soft-ripened Camembert-like molded cheese samples [6].

In this work, the potential of 2D ^1^H-NMR T_1_–T_2_ for inline cheese characterization was tested. In particular, we monitored the time evolution of Pecorino, a cheese variety produced in different regions of central and southern Italy from ewes’ milk, according to local or regional traditions differing in part from place to place [20,21]. Several Pecorino cheese samples, with different ripening degrees which had been stored for up to two years in a refrigerator under vacuum-packaging conditions, were analyzed with a portable and non-destructive surface-NMR instrument in a flexible, relatively inexpensive, and customizable way [22,23].

Before packaging, and at the end of the storage period, Differential Scanning Calorimetry (DSC) and Attenuated Total Reflectance Fourier-Transform Infrared Spectroscopy (ATR-FTIR) measurements were performed on the same cheese samples. Since these two techniques are recognized as invaluable tools in cheese control, they can be used as benchmarks for the NMR results. ASCA, analysis of variance (ANOVA) simultaneous component analysis (SCA), was applied to the NMR maps and the profiles obtained from DSC and ATR-FTIR to compare their statistical analysis over ripening and storage time.

## 2. Results and Discussion

### 2.1. The 2D ^1^H-NMR Relaxometry

**Figure 1:**
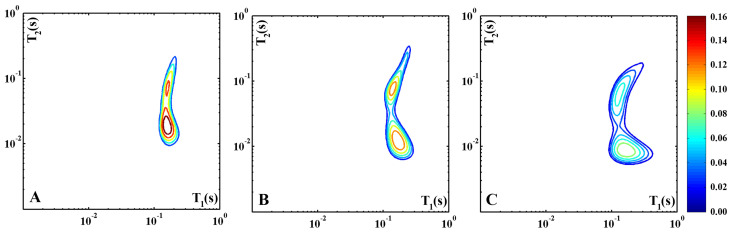
Three 2D ^1^H-NMR T_1_–T_2_ maps of a Pecorino cheese sample recorded with different values of ${2\tau }_{E}$: 100 µs (**A**), 300 µs (**B**), and 500 µs (**C**). The color-scale bar is set to the highest intensity of the three maps, and the contours are equally spaced from 10% to 90% of the maximum intensity.

Figure 1 shows the T_1_–T_2_ correlation maps recorded from a slice of Pecorino cheese with the following ${2\tau }_{E}:\;$100 µs (Figure 1A), 300 µs (Figure 1B), and 500 µs (Figure 1C). In this figure, as in the following ones, the color-scale bar was set to the highest intensity of the shown maps, and the contours are equally spaced from 10% to 90% of the maximum intensity.

In Figure 1A, the correlation map depicts a proton signal distribution trend, typical of diary-based materials, made up of two large proton domains characterized by different T_2_ values (~10 ms and ~100 ms) but with similar T_1_ values ~ 100 ms. The bimodal distribution arises from the superposition of a relatively sharp peak characterized by T_1_/T_2_ ~ 10 and a broader feature with T_1_/T_2_ ~ 1. As introduced before, the T_1_ over T_2_ ratio depends on the intra- and inter-magnetic interactions and the self-diffusion regime experienced by protons pertaining to the domain. Unlike the solid fat and protein signals that decay too quickly (T_2_ ~ 100 µs) to be evaluated in low-resolution experiments, the liquid fractions of fat and water are both well detectable and can be distinguished because of their different molecular self-diffusion coefficients. Generally, this distinction is achieved by diffusion–transverse relaxation time correlation maps [19]. In this case, we collected T_1_–T_2_ correlation maps contrasted in diffusion by acquiring the correlated NMR signals with different echo times. In fact, due to the inhomogeneous magnetic field applied across the sample of the single-sided NMR device, T_2_, unlike T_1_, suffers from a further dephasing term due to diffusion in the resulting magnetic field gradient of the instrument. Then, to investigate the influence of the diffusion processes on the two proton populations in the map of Figure 1A, T_1_–T_2_ measurements were also performed with ${2\tau }_{E}\;$ = 300 μs (Figure 1B) and ${2\tau }_{E}\;$ = 500 μs (Figure 1C), i.e., with longer diffusion times. Unlike the domain with the smaller T_1_/T_2_ ratio, the peak characterized by the higher one evolves with increasing echo times as T_1_/T_2_ = 8 for ${2\tau }_{E}\;$ = 100 μs, T_1_/T_2_ = 15 for 2$\tau_{E}\;$ = 300 μs, and T_1_/T_2_ = 20 for 2$\tau_{E}\;$= 500 μs. As one should expect the shifts are due to a T_2_ decrease, while T_1_ remains unchanged. This evidence suggests that the domain with T_1_/T_2_ ~ 10 is characterized by a faster proton diffusion than that with T_1_/T_2_ ~ 1; therefore, the first peak can be assigned to water and the latter to fat molecules.

The observed mean relaxation times associated with water (T_1_ ~ 100 ms and T_2_ ~ 10 ms) are significantly lower than the bulk-water values (T_1_ = T_2_ ~ 1 s), indicating that the water molecules are strictly confined in the casein network. The sharp distribution of this proton population suggests that all the detected water molecules are in a fast exchange regime with each other. Differently, the relatively broader T_1_–T_2_ distribution of the fat phase points out a greater inhomogeneity in the dynamic behavior of the fat molecules, which can be related to the wide differences in the length of the aliphatic chains bound to glycerol, the coexistence of free fatty acids and triglycerides and the size distribution of the domains inside the protein network occupied by the fat molecules. The above assignment can assist with the interpretation of the 2D ^1^H-NMR T_1_–T_2_ data reported below, all recorded with 2$\tau_{E}\;$ = 300 μs.

**Figure 2:**
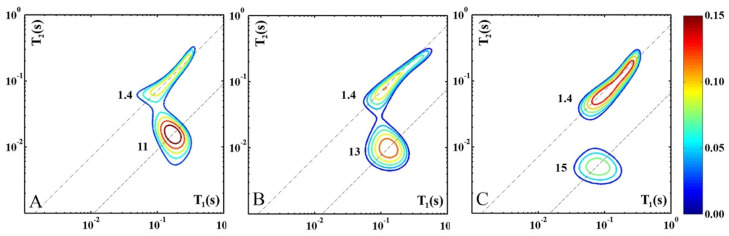
Three 2D ^1^H-NMR T_1_–T_2_ maps of *hm-0y* (**A**), *mm-0y* (**B**), and *lm-0y* (**C**) representative cheese samples characterized, respectively, by high-, medium, and low-moisture content, analyzed soon after purchase (*0 years* of storage time). For each proton domain the slope of the dashed line indicates the corresponding T_1_/T_2_ value.

Figure 2 shows the modifications occurring in cheese samples characterized by high-, medium-, and low-moisture content, analyzed soon after they were purchased (*0 years* of storage) and indicated, respectively, as *hm-0y* (Figure 2A), *mm-0y* (Figure 2B), and *lm-0y* (Figure 2C). This allows us to evaluate the changes in the contribution and dynamics of the protons of both water and fat molecules as ripening progresses. For each category of cheese, the map of a single specimen is shown as it is representative of all the samples.

The figure shows a progressive increase in fat content as the ripening degree increases, while the contribution of the water protons progressively decreases. The latter effect directly results from the cheese dehydration occurring in the ripening process. The apparent increase in the fat signal is also related to dehydration because the moisture reduction has a concentrating effect on the other cheese constituents, as described in the literature for different cheese varieties [24,25,26].

As ripening progresses the water signal shifts to higher T_1_/T_2_ ratios from 11 (*hm-0y*) to 13 (*mm-0y*) and up to 15 (*lm-0y*). This probably occurs due to a reduction in the volume of the cavities where water molecules are tightly confined. The size of the water clusters is primarily determined by the empty spaces within the protein network [6]. Therefore, the decrease in the relaxation times of water protons is a direct result of the shrinking protein network. The mobility of water molecules within the cheese sample is also affected by the increase in the water population engaged in polar interactions with peptides, salts, and hydrophilic organic compounds generated by the biochemical transformation of the cheese constituents [5]. The combination of these factors results in the greater immobilization of water molecules in hard cheeses compared to soft ones.

**Figure 3:**
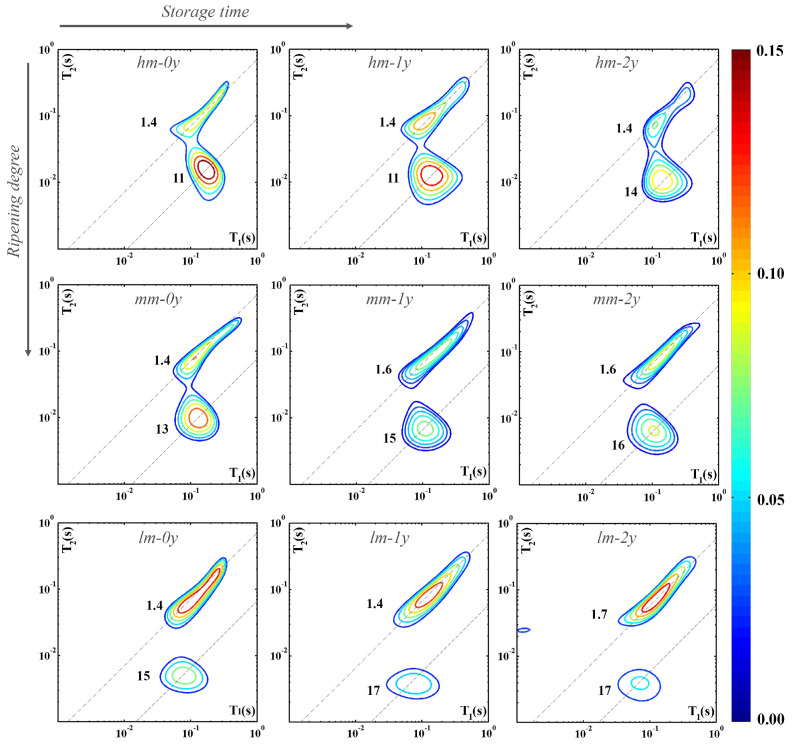
Nine 2D ^1^H-NMR T_1_–T_2_ maps of representative cheese samples with different moisture contents measured at different storage times. For each proton domain the slope of the dashed line indicates the corresponding T_1_/T_2_ value.

Figure 3 summarizes the modifications occurring in cheese samples *hm*, *mm*, and *lm* analyzed soon after the purchase (*0y*, first column), after one year (*1y*, second column), or after two years of storage (*2y*, third column). This allows us to evaluate the changes in the contribution and dynamics of protons of both water and fat molecules as the ripening degree (along the columns) and storage time (along the rows) progress. For each category of cheese, *hm*, *mm*, or *lm*, the map of a single specimen is shown as it is representative of all the samples.

As shown in the figure, the intensity of the distribution function related to the total amount of protons decreases as the ripening (see along the first column) and storage (see along the rows) factors increase. As storage time increases for the *hm*, *mm*, and *lm* samples, the trend of the signals attributed to the fat and water protons (lower distributions) seems to follow that observed as ripening progresses. In the cheese samples at the time of purchase with high (*hm-0y*) and medium moisture content (*mm-0y*), the fat-to-water ratio results in favor of the water content. Conversely, the contribution of fat protons in highly ripened cheese samples at the beginning of the storage (*lm-0y*) is noticeably higher than that of water. In any way, for the three samples *hm*, *mm*, and *lm*, the fat-to-water ratio increases as the ripening and storage factors increase.

Comparison of the different panels of Figure 3 also reveals that while the signals attributed to water and fat are partially superimposed in soft cheese and become more and more separated with the progress of ripening, these contributions tend to move away by also increasing the storage time. One must remember that a wide range of relatively small organic molecules are generated by proteolysis (short peptides and amino acids), lipolysis (free fatty acids and volatile compounds), and glycolysis (lactate and short-chain carboxylic acids) of the cheese constituents. Signals of the protons of these molecules, although not exchangeable, may interfere with those of water under the condition that the related relaxation times become similar [5].

Moreover, the increase in the storage time induces an increase in the T_1_/T_2_ ratio of the water proton component from 11 (*hm-0y* and *hm-1y*) to 14 (*hm-2y*) for the *hm* representative sample (see first row of Figure 3). Similarly, the second row highlights an increase in the T_1_/T_2_ ratio of the same component of the *mm* representative sample, from 13 (*mm-0y*) to 15 (*mm-1y*) and up to 16 (*lm-2y*). Finally, in the last row of the same figure, an increase in the T_1_/T_2_ ratio from 15 (*lm-0y*) to 17 (*lm-1y* and *lm-2y*) for the *lm* representative sample is evident. This suggests that long storage under a vacuum and in a refrigerator causes a greater immobilization of water molecules in cheese samples characterized by different ripening times at the time of purchase.

All these findings show that biological transformations of the cheese constituents continue in the stored vacuum-packaged samples, although the pattern of proton signals seems to evolve differently under the effects of ripening in farm conditions and in storage under vacuum-packaging conditions.

The ANOVA-like partitioning was applied to the T_1_/T_2_ ratio projections from the 2D spectra. At the time of processing, two out of fourteen NMR data sets were found to be partially unreadable and therefore unusable. As a consequence, the analysis of the NMR data concerns twelve samples (four *hm*, five *mm*, and three *lm*) measured at three different times for a total of thirty-six data sets.

By applying ASCA to the NMR data, two simultaneous components (SC1 and SC2) could be extracted for each factor as the effects of all of the three initial ripening degrees were investigated for as many storage times. The analysis indicated that both main effects were statistically significant, whereas their interaction effect was not. The loadings $P_{X_{storage}}$ and $P_{X_{ripening}}$ were also investigated to evaluate which spectral features are mainly responsible for the differentiation of the samples stored for a different amount of storage time and with different moisture content.

**Figure 4:**
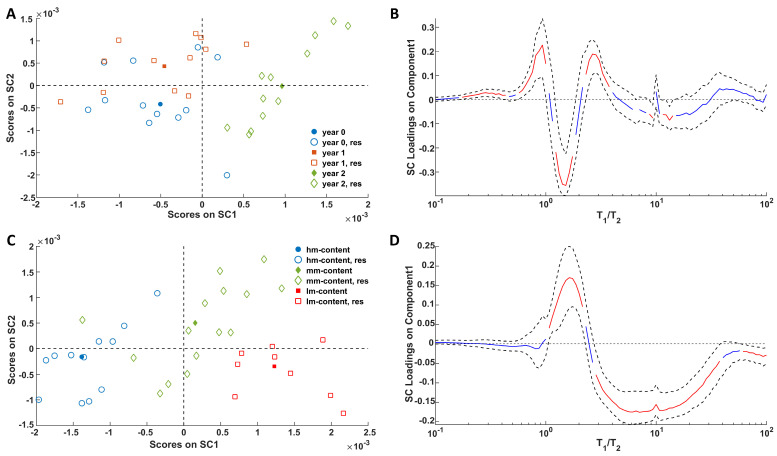
SCA modeling of the storage (**A**) and ripening (**C**) effects on NMR T_1_/T_2_ distribution maps. For the score plots, full symbols represent the level averages (i.e., the centroid) and empty symbols represent the residuals. Loadings of the NMR signals on SC1 of the storage (**B**) and ripening (**D**) effects together with their bootstrapped confidence intervals (dashed black lines). Loadings are highlighted in red when significant; otherwise, they are in blue.

In Figure 4 the plots of the back-projected scores (Figure 4A,C) associated with storage and ripening, respectively, are shown. With regard to the storage effect, SC1 explains 79.4% of the variance, while SC2 accounts for the remaining 20.6%. For the ripening effect, SC1 and SC2 explain 87.8% and 12.2% of the variance, respectively.

Figure 4A shows a clear separation between the acquisitions from samples stored for 2 years and those stored for 0 or 1 year, which partially overlap. The distribution of the samples in the SC space (Figure 4C) further highlights the significant effect of ripening—an effect even more pronounced than that of storage. In this case, SC1 effectively captures the differences among the three groups. To better understand how these trends relate to changes in the relaxation profiles, the loadings on SC1 can be examined in Figure 4B,D. Notably, the fat domain (characterized by a T1/T2 ratio of ~1.4 and higher absolute loading values in Figure 4B) is the most clearly impacted by the aging of Pecorino cheese over the two-year storage period. In this regard, it must be noted that long storage causes a decrease in the fat component with a T_1_/T_2_ ratio close to 1.4 and a simultaneous increase in the components characterized by both T_1_/T_2_ values lower and higher (in any case the T_1_/T_2_ ratio has to be greater than the physical limit, i.e., T_1_/T_2_ > 1). Conversely, the whole network of fat and water in cheese is highly influenced by the different extents of ripening. The progress of ripening is associated with a decrease in the water contribution and an increase in the fat component. It is also interesting to note that the significant effects attributed to water protons cover a relatively wide T_1_/T_2_ range. This can be explained by the coexistence of water molecules experiencing different degrees of immobilization due to the different sizes of the domains in which these are confined and/or the different strengths in the interactions with the polar cheese components. Therefore, looking at the ASCA results it seems that the proposed method is capable of recognizing cheese samples stored for different amounts of time regardless of their different initial degree of maturation (a key factor in food preservation), and vice versa.

### 2.2. DSC Analysis

**Figure 5:**
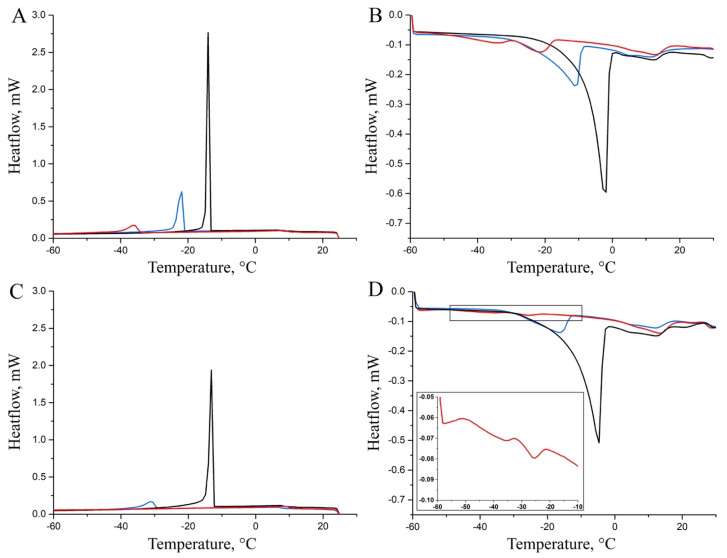
DSC cooling (**A**–**C**) and heating (**B**–**D**) thermograms acquired at *0* (**A**,**B**) and *2 years* (**C**,**D**) of storage time on representative *hm* (black line), *mm* (blue line), and *lm* (red line) cheese samples.

The DSC cooling and heating curves, collected on *hm*, *mm*, and *lm* samples at *0 years* and *2 years* of storage and reported in Figure 5, are dominated by the peaks derived from the freezing and melting of water. The cooling rate to the crystallization temperature affects the polymorphic composition of the crystallites: faster cooling results in a lower proportion of the stable, high-melting form.

The low-intensity transition, observed between 0 °C and 25 °C when both fresh (*0 years*) and stored (*2 years*) Pecorino cheeses were heated, could be attributed to the fat content [27]. However, the low intensity and broad shape of these signals prevent clear observations of the effects of ripening or storage on the fat component of the samples. Table 1 presents data on the transitions of the aqueous phase observed during both cooling and heating steps, i.e., the integral values of the transitions and their onset temperatures.

The thermograms acquired by cooling the Pecorino cheese samples soon after their purchase (Figure 5A) are characterized by a single and well-defined exothermic transition. The intensity of the freezing transitions decreases significantly with the degree of maturation from *hm* to *mm* to *lm* samples, as shown in Table 1, first column, which is where the water content of the samples is indicated. Similar results are observed by comparing the integrals of the melting transitions (Figure 5B and Table 1, third column). However, heating the most ripened sample results in two transitions in the thermogram, which may be explained by the presence of two distinct water domains confined within the cheese matrix. Additionally, Table 1 (second and fourth columns) shows that the onset temperatures for the freezing and melting transitions of water decrease with increased ripening. This effect can be attributed to the confinement of the aqueous phase into smaller domains and a higher proportion of water molecules interacting with polypeptides and polar compounds as ripening progresses. A further potential contribution to this effect is the decrease in the water/solute ratio due to the concentration of salts and solubilization in the aqueous environment of polar organic compounds generated by maturation processes.

The trend of the thermograms collected from the *hm-2y*, *mm-2y*, and *lm-2y* samples (Figure 5C,D) is qualitatively similar to that observed for the samples with different ripening degrees analyzed soon after the purchase (*hm-0y*, *mm-0y*, and *lm-0y* in Figure 5A,B), although the signals are less intense. Based on this general trend, no transition is actually observed for the *lm-2y* samples during the cooling ramp, while two low-intensity melting transitions are detected for these specimens (inset of Figure 5D). A comparison of the integral values for the transitions of the cheese samples with the same ripening degree before and after a two-year storage time suggests a loss of water that can be ascribed to a partial permeability of the packaging material [28]. It must also be remarked that DSC measurements on the stored samples require the removal of the packaging. As also described in the literature [29,30,31], this procedure resulted in the loss of a thin, visible layer of liquid located between the surface of the sample and the plastic packaging. Nevertheless, DSC results show that the packaging preserves much of the original moisture in the differently ripened cheeses even after two years of storage. In contrast, the ripening process that takes place on the farm before selling leads to a quicker dehydration of the Pecorino cheese. Inspection of Table 1 also reveals a general decrease in the onset temperature of the stored samples compared to those analyzed shortly after purchase, likely due to the progression of the aforementioned processes (hydration of polypeptides, concentration of salts, and solubilization of polar compounds) leading to an increase in the solute/water ratio. As described for different cheese varieties [30,31,32], although vacuum-packaging and storage in the refrigerator (3–9 months) can partially stabilize the gross compositional characteristics of cheeses, biological transformations still take place.

ASCA was conducted on DSC data using a methodology consistent with what has been previously described for NMR data. Two SCs (SC1 and SC2) could be extracted for the ripening factor, but only one SC (SC1) could be extracted for the storage time since the latter was studied considering only two levels, i.e., *0 years* and *2 years*.

The analysis revealed that both storage and ripening are statistically significant factors. The explained variance for $X_{storage}$ and $X_{ripening}$ was 100.0% and 93.2% of the total data variance, respectively.

**Figure 6:**
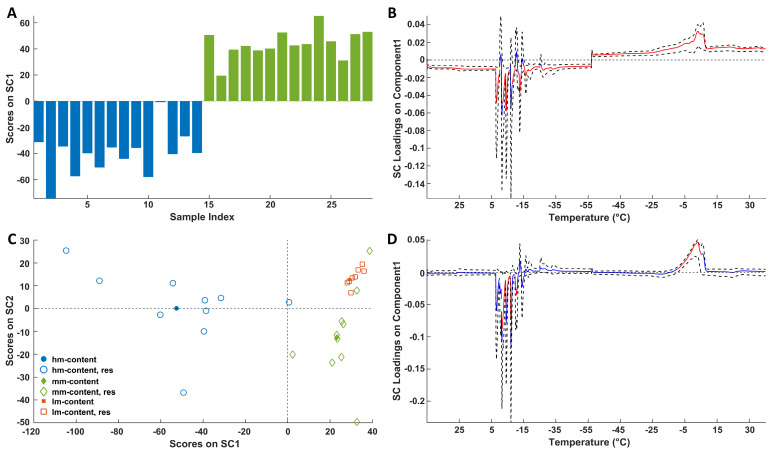
SCA modeling of the storage (**A**) and ripening (**C**) effects on DSC thermal profiles. In (**C**) full symbols represent centroids. Blue bars represent 0 year, whereas green bars refer to 2 years. Loadings of the DSC signals on SC1 of the storage (**B**) and ripening (**D**) effects together with their bootstrapped confidence intervals (dashed black lines). Loadings are highlighted in red when significant; otherwise, they are in blue.

The scores plots linked to the storage (Figure 6A) and ripening (Figure 6C) reveal distinct groupings of samples across different storage times and maturation stages.

Only two levels of storage time (*0* and *2 years*) were available. Notably, Figure 6A reveals a clear trend between that samples at *0 years* (at negative SC1 values) and those at *2 years* (at positive SC1 values).

Figure 6C shows group tendencies according to the ripening. Indeed, the *hm* cheese group presents negative scores for SC1 contrarily to medium-hard (*mm*) and hard (*lm*) samples (overlapped at positive values of SC1). These latter samples (*mm* and *lm*) show different trends along SC2; in fact, *lm* samples tend to fall at positive values of this component, whereas *mm* objects present negative SC2 values. Regarding the loadings (Figure 6B,D) it must be remembered that the leftmost (−15 °C) and the rightmost (−5 °C) signals arise from the exothermic freezing and endothermic melting of water, respectively. Looking at the loadings it is possible to conclude that discrimination of soft and ripened samples (*0 years*), on the one hand, and fresh (*0 years*) and stored (*2 years*) cheese samples, on the other hand, is always related to a decrease in the water content in the samples. Interestingly, the temperature range of the significant effects around −15 °C (cooling) and −5 °C (heating) is wider when storage is considered compared to ripening. The wider inhomogeneity of the thermal behavior of water in the stored cheese samples could be the consequence of a greater polydispersity in the water/solute ratios.

### 2.3. ATR-FTIR Characterization

**Figure 7:**
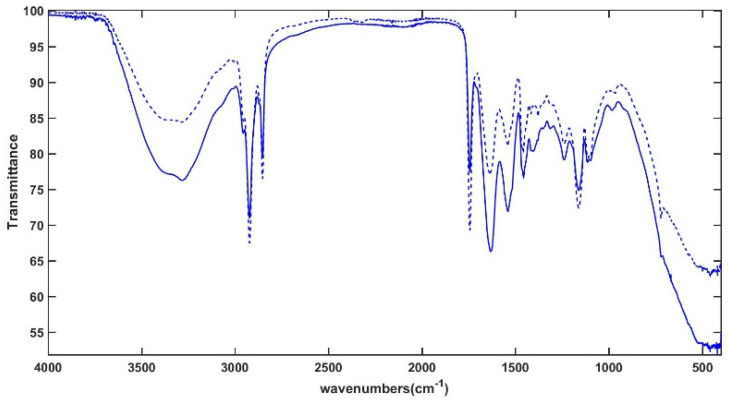
Mean ATR-FTIR spectra of mid-moisture cheese samples at *0 years* (*mm-0y*, dashed line) and *2 years* (*mm-2y*, full line) of storage time.

Figure 7 displays the mean ATR-FTIR spectra of *mm* samples collected at *0 years* (dashed line) and *2 years* (full line) of storage. The signals, which reflect the typical vibration patterns of the main cheese constituents (proteins, fats, carbohydrates, and water), were assigned consistently with previous ATR-FTIR investigations of dairy products [33].

The broad band between 3600 and 3000 cm^−1^ is mainly related to O–H stretching of water, but hydroxyl stretching of alcoholic groups in sugars and N–H vibrations of proteins and amino acids also contribute to this signal. The vibrational modes of C–H bonds, mainly of long-chain fatty acids, are responsible for the sharp signals at 2922 and 2851 cm^−1^ (antisymmetric and symmetric CH_2_ stretching modes, respectively) and the weak absorptions at 2941 and 2848 cm^−1^ (asymmetric and symmetric stretching vibrations of the terminal CH_3_ groups, respectively). The sharp band centered at 1742 cm^−1^ is generated by the carbonyl stretching of triacylglycerols and carboxylic acids. The broad signals at 1629 cm^−1^ and 1539 cm^−1^ originate from typical Amide I and Amide II vibrations of proteins, respectively, which are sensitive to changes in their secondary structure, protein aggregation, and protein/water interaction [34]. The bending of water molecules also falls into the same spectral range. Several vibrations of lipids, organic acids, amino acids, and carbohydrate derivatives can be detected in the so-called “fingerprint region”, including the bending modes of O–C–H, C–C–H, and C–O–H (1500–1200 cm^−1^) and the C–C and C–O stretching modes (1153–900 cm^−1^). In particular, the signals around 1456 and 1379 cm^−1^ originate from C–H bending in methylene and methyl groups and the signal at 1414 cm^−1^ results from the rocking vibrations of C-H bonds of cis-disubstituted olefins. The bands at 1239, 1163, 1107, and 1097 cm^−1^ arise from C-O vibrational modes of the ester linkage in triacylglycerols, but C–O stretching modes in lactose and monosaccharides also contribute to the absorption at 1163 cm^−1^. The sharp signal recorded at 722 cm^−1^ can be assigned to the –CH_2_ rocking vibrations related to the hexagonal packing of triacylglycerols in crystallized fat [35]. The qualitative comparison of the spectra displayed in Figure 7 reveals a strong reinforcement of the absorption at 1400 cm^−1^ in the stored samples compared to the fresh ones. This band can be attributed to carboxylate ions [36] and can be associated with the transformation of lactose, the main milk carbohydrate, first to lactate and then to propionate, acetate, formate, and pyruvate, depending on the microorganisms involved [4].

ASCA was performed on the spectra using a methodology similar to that described earlier. Moreover, as for DSC analysis, by applying ASCA to the ATR-FTIR data two SCs (SC1 and SC2) for the ripening factor and one (SC1) for the storage time could be extracted.

The results indicated that storage and ripening are statistically significant effects. The variance explained by the models is 100.0% for $X_{storage}$ and 96.4% for $X_{ripening}$.

**Figure 8:**
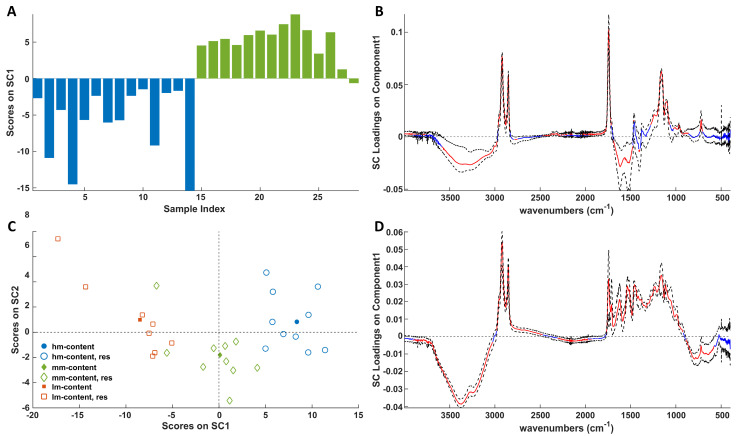
SCA modeling of the storage (**A**) and ripening (**C**) effects on ATR-FTIR spectra. In (**C**), full symbols represent centroids and empty symbols represent the residuals. Blue bars represent *0 year*, whereas green bars refer to *2 years*. Loadings of the IR signals on SC1 of the storage (**B**) and ripening (**D**) effects together with their bootstrapped confidence intervals (dashed black lines). Loadings are highlighted in red when significant; otherwise, they are in blue.

The score plots associated with storage (Figure 8A) and ripening (Figure 8C) effects delineate discernible patterns among samples across various storage times and stages of maturation. Notably, Figure 8A demonstrates that samples with *0 years* of storage time exhibit negative SC1 values, while those with *2 years* display positive values. Conversely, in Figure 8C, the distribution of *lm*, *mm*, and *hm* samples along SC1 progresses from negative to positive scores, respectively, indicating that ripening and maturation effects are oriented along opposite SC1 directions. The investigation of the loadings (Figure 8B,D) reveals that positive and negative loadings in Figure 8C are associated with a decrease or increase, respectively, of the vibration-active chemical moieties, whereas the loadings shown in Figure 8D must be interpreted oppositely. Figure 8D reveals that ripening produces a decrease in water content, as highlighted by the OH stretching band and an increase in all the cheese macronutrients, namely fats, proteins, and carbohydrates. It follows that dehydration and the consequent concentration of the other cheese components, as widely documented in the literature for several cheese varieties [24,25,26] and discussed before, is the major effect of Pecorino cheese ripening on the infrared spectra. On the other hand, storage causes a significant decrease in the absorption of fats and sugars, which is diagnostic of extensive lipolysis and glycolysis. Regarding signals in the spectral range 1700–1500 cm^−1^, storage produces an absorbance increase. It must be remarked that in this region, apart from Amide I and Amide II vibrations of proteins, the bending mode of water also contributes. According to the literature [37,38], the evolution of the Amide I and Amide II bands with ripening is rather variable depending on the type of cheese and the maturity level [36,39]. Nevertheless, an increase in both bands as ripening progresses, which has been associated with proteolytic activity, has been observed in the maturation of Camembert-type cheeses [36] and goat cheeses [40]. Also significant is the increase in the absorption band related to carboxylates (1400 cm^−1^) derived from lactose degradation. The apparent increase in moisture content, suggested by the negative loadings in the 3600–3000 cm^−1^ range, is in contrast to the DSC results and the evidence of a loss of water upon the removal of the plastic packaging before both DSC and ATR-FTIR measurements. Nevertheless, in ATR-FTIR measurements the cheese sample is pressed to favor its contact with the ATR crystal. In this condition, as also described by other authors [41], seepage of the liquids entrapped inside the cheese can slightly modify the composition at the cheese surface, leading to the overestimation of the moisture content. Another interesting effect is that ascribable to structural changes in the fat matrix of Pecorino cheese, which is highlighted by the significant increase and decrease in the signal of the CH_2_ rocking vibration mode (727 cm^−1^) with the progress of storage and ripening, respectively.

### 2.4. Final Discussion

Magnetic resonance relaxation time correlations in the proton signal, coupled with ASCA, were employed as a non-invasive and non-destructive approach to investigate the significance of storage time and ripening effects in Pecorino cheeses. More conventional techniques, such as DSC and ATR-FTIR, were also applied and likewise analyzed to support the NMR outcomes. ASCA suggests that the NMR approach can successfully distinguish between vacuum-packed cheeses stored for different durations (storage time) irrespective of their original moisture content (ripening degree), and vice versa. This evidence, supported by ASCA on DSC and ATR-FTIR data, reveals that observed changes in the composition of a cheese sample are influenced by a combination of both the maturation level at the time of purchase and the storage time.

Although a larger number of samples would be necessary to obtain quantitative insights into a proper sample classification, NMR relaxometry presents a valid alternative to traditional methods for studying the ripening and storage of Pecorino cheese. In addition to yielding results comparable to those of more conventional techniques, the proposed NMR approach is non-destructive, thereby preserving the integrity of the cheese during analysis. Furthermore, the maturation process could be examined using different RF coils with greater penetration depth without the need for coring or cutting the cheese wheels, which are instead indispensable steps prior to applying the other two benchmark techniques.

In summary, our findings represent a solid starting point for future investigations into the complex interplay between storage time and maturation in shaping the characteristics of dairy products, with a focus on the chemical and physical properties of their water and fat components. Future research will aim to expand the proposed methodology to a broader range of cheese types and to integrate the NMR-ANOVA approach with complementary analytical techniques. This will enable a more comprehensive investigation of the biochemical processes occurring in both the aqueous and lipid fractions of cheeses.

## 3. Materials and Methods

### 3.1. Pecorino Cheese Samples

The experiments were designed to evaluate the effect of the following two factors: the *ripening* degree of the cheese samples at the time of purchase (February 2019) and the *storage* time within the following two years, during which the vacuum-packaged samples were kept in a refrigerator. The study was conducted on fourteen cheese samples from various Pecorino varieties, each with different maturation levels based on the commercial labeling. Pecorino typically matures in 20–60 days for soft cheeses and 8–12 months or more for hard ones [21,42]. The sampling was primarily aimed at representing the different typologies of commercial products according to the maturation stage, i.e., soft, semi-hard and hard cheeses. However, to limit the variability related to the cheese-making process and the origin of the raw materials, all the Pecorino cheese samples analyzed were produced in the internal Apennine area of Abruzzo (central Italy), using raw sheep milk and animal rennet as coagulant.

The moisture content at the time of purchase of the investigated Pecorino cheese was experimentally evaluated by the weight loss observed after a 24-h drying process conducted on small specimens with a Büchi TO-51 (Büchi, Switzerland) glass oven. Based on the ripening degree stated on the label and the measured moisture content values, the study was conducted on the following:

- Five soft-ripening cheese samples with a high-moisture content (>30%);
- Five medium-ripening cheese samples with a mid-moisture content (22–30%);
- Four hard-ripening cheese samples characterized by a low-moisture content (<22%);

Hereafter referred to as *hm*, *mm*, and *lm*, respectively.

For the NMR experiments, whole slices (weight ~ 200 g) of the selected cheese samples were placed in polyethylene film bags (thickness ~ 100 μm), vacuum-packaged using a domestic vacuum packaging machine, and stored in a LCexv 4010 MediLine (Liebherr, Austria) refrigerator at 4.0 ± 0.1 °C.

NMR signals were acquired from the packaged samples within 10 days of purchase and after one and two years of storage under the previously described conditions, referred to as *0*, *1*, and *2 years*. Differently, DSC and ATR-FTIR measurements were taken on small and unpackaged cheese samples only at the beginning and at the end of the full storage period, specifically at *0 and 2 years*. The decision to not collect ATR-FTIR and DSC measurements after one year was based on the need to preserve the storage conditions for the NMR experimental setup throughout the two-years observation period, ensuring that the packaging remained sealed during the intermediate period.

### 3.2. The 2D ^1^H-NMR T_1_–T_2_

**Figure 9:**
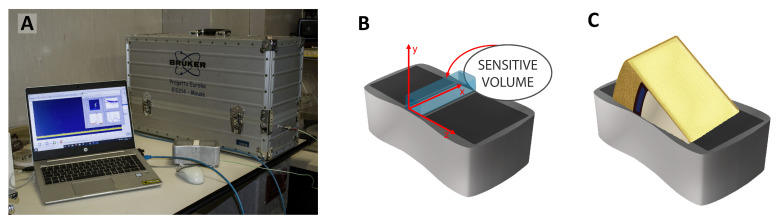
(**A**) NMR single-sided apparatus, (**B**) sensitive volume, (**C**) sample arrangement on the surface probe.

The 2D ^1^H-NMR T_1_–T_2_ experiments were carried out using a single-sided NMR probe (mq-ProFiler, Bruker Biospin, Italy) that works at a Larmor frequency of 17.8 MHz for protons. The experimental setup (schematized in Figure 9A) offers the chance of a non-invasive and non-destructive approach to also detect ^1^H-NMR signals from very large samples [43]. The sensitive volume of the surface probe (x, y, z) of about 2 cm × 0.2 cm× 0.8 cm (Figure 9B) enables it to excite the sample surface up to a depth of about 0.2 cm. Moreover, since the ^1^H-NMR signal is proportional to the total amount of proton content within the sensitive volume [43], for a sample larger than the surface probe, as it is for our samples, the signal amplitude allows for the evaluation of the volume fraction occupied by protons.

In each measurement the entire vacuum-packed cheese slice was placed on the probe (Figure 9C). It was verified that the packaging material does not contribute to the NMR signal. Moreover, the thickness of the plastic film bags of just 100 μm ensures that the 2 mm penetration depth of the NMR probe is such that the NMR signal comes exclusively from the slice of cheese under investigation.

The correlated relaxation data was collected using a radiofrequency pulse sequence during which the spin system evolves under the T_1_ (evolution period) and the T_2_ relaxation mechanisms (detection period) [42]. Relaxation data were acquired with a saturation recovery and a Carr–Purcell–Meiboom–Gill (CPMG) pulse sequence described as ${\left[saturation-\tau_{S}-\pi /2-\tau_{E}{\left(\pi -\tau_{E}-acquisition-\tau_{E}-\right)}_{nE}-\tau_{RD}\right]}_{m}$. Saturation represents ten *π*/2 pulses of fixed length, while *π*/2 and *π* indicate the standard radio-frequency pulse. The T_2_ encoding time, $\tau_{E}$, was established for different T_1_ encoding times, $\tau_{S}$. The total T_2_ encoding time, $2\tau_{E}n_{E}$, was set to ~600 ms with 2$\tau_{E}\;$ = 300 μs and $n_{E}=2000$, whereas the T_1_ encoding was performed ranging $\tau_{S}$ from 1 ms to 10 s, according to a geometric progression with $n_{S}=51$ different values. Finally, the recycling delay time, $\tau_{RD}$, was set to 2 s and each signal acquisition was averaged over m = 64 scans. The 2D Fast Laplace inversion algorithm [18] was used to process the data matrix, $n_{E}\times n_{S}$, to obtain the density distribution function of the relaxation times, f(T_1_, T_2_).

For each comparison of data, the reported signal density distribution maps obtained from cheese samples with different ripening and/or storing time were normalized to their absolute maximum value.

### 3.3. DSC

A Mettler Toledo DSC-3 (Mettler Toledo, Columbus, OH, USA) differential scanning calorimeter equipped with a Mettler Toledo™ TC100 MT Intracooler was used to evaluate the thermal properties of the Pecorino cheese samples. Indium (melting point at 156 °C) and water (melting point at 0 °C) were used as standards to calibrate the DSC calorimeter for both cooling and heating scans. The cheese samples (20 mg) were hermetically sealed in 40 µL aluminum crucibles with a pierced lid. The reference was an empty hermetically sealed aluminum pan with a pierced lid of the same size. The sample was placed in the calorimeter at room temperature and subjected to at first cooling from 25 to −60 °C, followed by a heating step from −60 to 30 °C, each performed at a rate of ±2 °C/min. Nitrogen (99.99% purity) was used as a purge (50 mL/min) and dry (200–250 mL/min) gas to pressurize and maintain an inert atmosphere inside and outside the furnace chamber, respectively. Under the experimental conditions, reproducible thermal recordings were obtained, i.e., the uncertainty about the temperatures was ±0.3 °C. The raw DSC profiles were exported from the STAR^e^ Evaluation Software V16.10 (Mettler Toledo, Columbus, OH, USA) and imported into Matlab (R2020b; The Mathworks, Natick, MA, USA) to be further processed.

### 3.4. ATR-FTIR

Infrared spectra of Pecorino cheese slices were acquired employing a PerkinElmer Spectrum Two™ FTIR spectrometer (PerkinElmer, Waltham, MA, USA), equipped with a deuterated triglycine sulfate (DTGS) detector and a Universal ATR (uATR) accessory featuring a single-bounce diamond crystal. Spectra were recorded over the 4000–400 cm^−1^ range with a resolution of 1 cm^−1^. Pressure was adjusted via the instrument’s built-in monitoring system to optimize spectral intensity. For each replicate, twelve scans were averaged. Before each acquisition, the ATR crystal was cleaned with methanol and air-dried. The resulting ATR-FTIR spectra were exported using Spectrum software (https://spectrum-software.com.au/) (PerkinElmer) and imported into MATLAB (https://www.mathworks.com/products/matlab.html) for further analysis.

### 3.5. ASCA

ASCA is a multivariate method that combines the principles of ANOVA (analysis of variance) and PCA (principal component analysis) to analyze complex experimental designs [44]. It decomposes the variation in a data set into components that reflect the effects of controlled factors (in this study, storage time, ripening degree, and their interactions).

Considering an experimental data matrix X, ASCA decomposes the mean-centered matrix ($X_{c}$) according to Equation (1) as follows:

(1) $$X_{c}=X_{storage}+X_{ripening}+X_{storage*ripening}+X_{e}$$

where $X_{storage}$ accounts for the effect of storage time, $X_{ripening}$ for the effect of ripening degree, $X_{storage*ripening}$ is the interaction between the two investigated effects, and $X_{e}$ accounts for the residual (unexplained) variance.

To test the significance of each effect, the sum of squares (SSQ) of each effect matrix is calculated and compared to a reference distribution obtained by permutation testing (10,000 iterations).

Each effect matrix is then analyzed using simultaneous component analysis (SCA), which identifies the main sources of variation within each factor. The first component (SC1) explains the largest part of the variance, followed by the next components in decreasing order [45].

A modified score plot [46] is used to visualize both systematic variation and random variability. Additionally, a bootstrap procedure is applied to estimate confidence intervals for the loadings, providing insight into the robustness of the observed patterns.

## Figures and Tables

**Table 1:** Integral and onset temperature mean values of the transitions revealed during the cooling and heating of the *hm*, *mm*, and *lm* cheese samples at *0 years* and *2 years* of storage time.

	Cooling		Heating			
	Integral (mJ)	Onset T (°C)	Integral (mJ)		Onset T (°C)	
hm-0y	1509	−13.4	−1534		−8.8	
mm-0y	603	−21.0	−641		−22.4	
lm-0y	224	−34.1	−165	−64	−28.9	−38.1
hm-2y	838	−12.5	−845 −227		−13.0 −32.2	
mm-2y	174	−29.3				
lm-2y	−	−	−10	−8	−30.8	−8.6

## Data Availability

Data is available on request.
